# Online Sexual Partner Seeking as a Social Practice: Qualitative Evidence from the 4^th^ British National Survey of Sexual Attitudes and Lifestyles (Natsal-4)

**DOI:** 10.1080/00224499.2021.1994516

**Published:** 2021-11-18

**Authors:** David S Reid, Wendy G. Macdowall, Ruth Lewis, Bernie Hogan, Kirstin R. Mitchell, Raquel Bosó Pérez, Jo Gibbs, Clarissa Smith, Feona Attwood, Catherine H. Mercer, Pam Sonnenberg, Chris Bonell

**Affiliations:** a Department of Public Health, Environments and Society, London School of Hygiene & Tropical Medicine; b MRC/CSO Social and Public Health Sciences Unit, University of Glasgow; c Oxford Internet Institute, University of Oxford; d Institute for Global Health, University College London; e Department of Arts, Northumbria University; f Independent Scholar

## Abstract

Once perceived as a means for those unsuccessful at traditional dating, online dating has become normalized as a way to seek sexual or romantic partners. In 2019, we interviewed 40 British adults on the role of digital technologies in their sexual lives; this paper draws on the accounts of 22 who had used such technologies for seeking partners. We analyzed qualitative accounts of online partner seeking as a social practice, drawing on a sample diverse in age, gender and sexual orientation, and informed by sexual script and social practice theory. Our theoretically informed analysis emphasized the multiple meanings and goals involved, the affordances of the technology and individuals’ skills. Our study provided several novel contributions. Young heterosexual people commonly used general social media, rather than dating apps, to meet partners; meeting partners often involved complex interplays between online and offline networks and encounters. Risks were defined not merely in relation to “risky others” but in terms of one’s own actions or attitudes. Participants deployed various skills in minimizing harms such as non-consensual sharing of intimate images, and used self-care skills such as setting limits to engagement.

## Introduction

Advances in information and communication technologies have resulted in their widespread integration into everyday lives (Finkel et al., 2012), including sexual lives (Anderson et al., 2020). Technology is increasingly a setting for, and modality of, sexual interaction and expression (Hogan et al., 2011; Jung et al., 2019). Once perceived as a means for those unsuccessful at traditional dating (Anderson et al., 2020), online dating has become normalized as a way to seek sexual or romantic partners (Smith, 2013). Industry data suggest that a sixth of UK adult internet users visited a dating site or app during June 2019 alone, largely via smartphones (Comscore, 2019). In the USA, nearly a third of adults reported using a dating site or app and the most common place to meet new partners is now online (Anderson et al., 2020). Driven by advances in mobile technology, including geolocation (Jung et al., 2019), there has been a shift from the use of dating websites accessed via personal computers toward dating apps accessed via smartphones (Ward, 2017). A US survey found that just over half of users of one major app, Tinder, reported using this for dating and around a quarter for sexual encounters or “hook-ups” (Hobbs et al., 2017). More younger people and more men than women use Tinder in this way (Sumter et al., 2017). Other research has explored online dating as a gendered and racialized practice (Curington et al., 2015).

Public health research has mainly framed online partner seeking as a risk factor for negative sexual and mental health outcomes (Cabecinha et al., 2017; Couch et al., 2012). Finding partners online is associated with reporting various sexual risk behaviors (such as condomless sex and higher partner numbers) and, among men, sexually transmitted infection diagnoses and human immunodeficiency virus (HIV) testing (Cabecinha et al., 2017). Women report receiving verbal abuse and threats of violence from online contacts more commonly than do men (Anderson et al., 2020). Such research has generally not examined the positive aspects of online partner seeking, how it is experienced and what it means to users.

Qualitative research has provided a useful counterbalance by exploring users’ own accounts of online partner seeking and the benefits as well as the risks that can arise from this. Some such studies have explored the accounts of samples of users diverse in terms of gender, sexual orientation, age and technology used, but a key limitation is that these have tended to explore only certain aspects of online partner seeking. For example, studies have focused on benefits, identifying that online dating can increase users’ potential sexual networks, and the speed and convenience of communication with contacts (Couch & Liamputtong, 2008). Other research has explored users’ accounts of harms, which can include: increased sexual risk behavior; emotional harms; and encountering dangerous or untrustworthy people. In users’ accounts, risks were framed in terms of risky “others”; users assessed contacts online, and used text messaging and telephone calls before meeting partners, to determine suitability and minimize danger (Couch et al., 2012).

Some qualitative research, with samples diverse in terms of gender and sexual orientation, has explored some aspects of the meaning of online dating (Degen & Kleeberg-Niepage, 2020), finding that dating apps became integrated into participants’ everyday routines and practices. Participants understood online dating in terms of an economic logic of attempting to compete in a market place and maximize the productivity of their investment. Users are reported to balance the stress of online dating with it enabling the achievement of valued goals.

Another focus of research has been on the online presentation of self (Ellison et al., 2006) where users balanced the dual imperatives of self-promotion and honesty in presenting themselves online in order to get noticed while minimizing the risk of rejection when meeting a contact in person. Building on this study, Ellison et al. (2011) developed the concept that the profile can be conceived of as a “promise” by the user to the audience rather than an exact representation of the user’s offline presence.

Other qualitative research has focused on heterosexuals’ use of specific dating apps. One study explored the accounts of mainly heterosexual Dutch Tinder users, focusing on their goals, self-presentation and screening strategies (Ward, 2017). This revealed that users’ goals could change over time, and could encompass not only seeking partners but also entertainment and increasing self-esteem through positive feedback from contacts. Resonating with the above notion of self-presentation as a “promise,” users selected profile photos aiming to present an ideal yet authentic self. Individuals engaged in searches not only to find people they liked, but also for clues on how to present themselves to attract others like them. A qualitative study of heterosexual young women’s use of Tinder focused on how this has affected sexual scripts (Christensen, 2020). The authors found that use of Tinder tended to encourage fleeting, informal relationships through the game-like “swipe logic” which discourages careful selection of matches. These findings suggested that Tinder maintained heteronormative and patriarchal hookup scripts which position men in control. Where women attempted to depart from such scripts, this was often met with resistance or confusion from matches. Another study explored online interactions on dating sites between men and women and reported on two discourses of men’s abuse of women (Thompson, 2018). One was the “not hot enough” discourse, in which men denigrated women’s looks, and a second was the “missing discourse of consent” discourse, in which men made unsolicited and often aggressive sexual propositions which took no account of women’s own agency or desires.

Several qualitative studies have focused on sexual minority (by which we mean self-identified sexual orientation other than straight/heterosexual) individuals’ use of online dating apps. One study compared the accounts of gay men using Grindr with heterosexuals using Tinder in terms of communication styles (Licoppe, 2019). Gay men using Grindr were found in general to aim to employ brief, information-checking communications to organize rapid hookups without getting distracted into more personal interactions. In contrast, many heterosexual users on Tinder tended to use more “topically-rich” conversation styles in order to explore potential as dating partners. In a study involving focus groups with gay men and women on their use of hookup apps (Albury & Byron, 2016), some participants described using geo-locative apps in a playful way to develop a sense of belonging by seeing the presence of other same-sex attracted people in their area. Participants described their personal “rules” employed when interacting or exchanging images or deciding when to meet a new partner offline. The findings indicated the symbolic importance of each partner deleting dating apps among those developing more committed relationships.

It is clear from the above that, although some qualitative studies have explored certain aspects of online partner seeking as a meaningful social practice, studies have tended to focus on certain groups of users, certain technologies and certain aspects of the practice. Our own study aimed to address these gaps by undertaking a broader qualitative exploration of all aspects of the practice among a diverse sample of participants.

### Theoretical Frameworks

A number of theoretical frameworks would be useful to inform such a broader exploration of online partner seeking. Sexual script theory has previously been used to examine accounts of online dating (Christensen, 2020). Sexual scripts constitute the “operating syntax” that give social meanings to sexual desire and behavior (Simon & Gagnon, 1986). At the macro level, “cultural scenarios” are the terrain in which sexuality is enacted, informed by social institutions such as the mass media (Wiederman, 2015). Interpersonal scripting refers to the behavior enacted within a cultural scenario, with individuals engaging in “scriptwriting” by adapting the general scripts they have learned to specific encounters (Simon & Gagnon, 1986). Intrapsychic scripting concerns the psychological organization of desires, fantasies and behaviors, with individuals considering the rules and meanings involved in enacting interpersonal scripts. However, sexual scripts may be of limited use in exploring the non-sexual aspects of online partner seeking as well as in understanding the technological material basis of online partner seeking and the skills required.

Conceiving of online partner seeking as a “social practice” may address such limitations. This framework aims to understand social actions in terms of their material and symbolic elements, and their relationship with other practices (Blue et al., 2016). According to Shove et al. (2012), practices can be understood in terms of three key elements: meanings (the embodied understandings of the symbolic significance of the practice); materials (comprising the objects and infrastructures facilitating the practice); and competencies (the understandings and know-how needed to carry out the practice). For example, online dating can be understood in terms of: the technologies involved (e.g., mobile phones, specific apps) and how these shape the actions involved; the meanings that online dating have for participants (e.g., its purpose, what it suggests about those who use it); the specific practices actually involved (e.g., flirting, arranging meetings); and how these might be linked to other practices (e.g., traditional dating or broader web-browsing).

Informed by these ideas about sexual scripts and social practices, we aimed to offer insights into online partner seeking across a diverse group of participants, technologies and practices. We explored the shared social meanings our participants attached to online partner seeking, the materials that enabled this, and the competencies participants developed and deployed in performing it.

## Method

Natsal-4 is a multi-method study involving a cross-sectional survey and qualitative research on sexual behavior, attitudes and health. It involved a first phase of qualitative research to explore new areas not previously examined in past Natsal waves.

### Participants

We conducted 40 in-depth interviews between May and June 2019. Half of the interviews had a primary focus on the role of digital technologies in participants’ sexual lives and a secondary focus on sexual wellbeing. In the other half, the focus was reversed. A market research recruitment agency (propeller-research.co.uk) recruited participants from among adult residents of England, Scotland and Wales registered with the company as being available for interview, originally recruited via media advertisements. Our approach to sampling aimed to ensure that the accounts we drew on would vary according to factors likely, according to the existing literature, to influence experiences of online partner-seeking. The recruitment agency emailed potential participants, using quota sampling to ensure purposive variation in terms of age, gender, ethnicity, relationship status, sexual orientation (in terms of self-identification as gay, straight etc.) and area of residence/area deprivation, and sent the research team the e-mail contact details of those who consented to be contacted. The company do not share details about response rates to their e-mails to potential participants. All participants whose details were sent to the research team consented to be involved in the study. Table 1 presents the sample characteristics and indicates that 22 participants had experience of online partner-seeking.

**Table 1.** Sample characteristics.

Total		Sought partners online N (%) 22	Subsequently met off line N (%) 16	Not sought partners online N (%) 18
Gender	Women	11 (50)	7 (44)	9 (50)
	Men	11 (50)	9 (56)	9 (50)
Age	<20	3 (14)	1 (6)	2 (11)
	20s	12 (55)	8 (50)	5 (28)
	30s	7 (32)	7 (44)	3 (17)
	40s	0 (0)	0 (0)	4 (22)
	50+	0 (0)	0 (0)	4 (22)
Ethnicity	White British	20 (91)	14 (88)	14 (78)
	Asian/British/Pakistani/Indian	1 (5)	1 (6)	3 (17)
	Black/British/African	1 (5)	1 (6)	0 (0)
	Mixed ethnicity	0 (0)	0 (0)	1 (6)
Relationship Status	Single	14 (64)	8 (50)	4 (22)
	In a relationship	8 (36)	8 (50)	14 (78)
Sexual Orientation	Heterosexual/Straight	13 (59)	9 (56)	17 (94)
	Bisexual	4 (18)	2 (13)	0 (0)
	Lesbian	1 (5)	1 (6)	1 (6)
	Gay	4 (18)	4 (25)	0 (0)

### Procedure

LSHTM’s research ethics committee approved the study (reference 17,046 26/4/2019). Six researchers (diverse in terms of gender and sexual orientation) conducted face-to-face interviews in settings of the participants’ choice, generally in their homes or at university offices. In all but one interview, only the interviewer and participant were present (in one interview, a friend of the participant was also present at the participant’s request). Prior to the interview commencing, the researcher provided participants with a study information sheet and the opportunity to ask questions before seeking consent.

Interviews were semi-structured, with all researchers using a common guide with questions about participants’ biographies, current circumstances, experiences of sexual and intimate relationships, and use of digital media generally and in relation to sex and relationships (see Appendix). Prior to fieldwork, researchers discussed how they would consistently use this guide. Interviewers first asked a general question about what part digital media play in participants’ lives before probing specifically about whether they used digital media to meet partners and, if so, what type of media, sites or apps they used and how they used them. Interviewers probed for the motivations, meanings and feelings associated with meeting partners online, its role in their lives, and the perceived benefits and harms.

All interviews were audio-recorded with participants’ permission and transcribed by a specialist company with identifiers removed by the research team.

### Data Analysis

Transcripts and field notes were entered into NVivo 11 to facilitate thematic content analysis. Our analytical sample was the 22 participants who reported experience of online partner seeking. We developed a coding framework with a priori codes, informed by social practice theory, covering meanings, materials and skills. We then used in vivo coding to generate inductive codes elaborating on or adding to these, grounded in recurring themes in participant responses. Axial coding then identified cross-cutting themes. Two researchers independently coded all data and then compared their responses to develop an account which benefited from each set of codes.

## Results

Participants were aged 19–64 years. All reported some experience of sexual activity. Of the 40 interviewed, 22 had looked for partners online (two once; eight occasionally; 12 more often), of whom 16 reported then meeting in person (Table 1). Of these 22, three were under 20 years old, 11 were women (none identifying as non-binary or transgender), two were from Black, Asian or Minority Ethnic communities, 13 identified as heterosexual/straight and 14 were single. No participants aged 40+ reported seeking partners online; this was also less common among those from nonwhite ethnic groups (2/6 compared to 20/34 among white participants) or in relationships (8/22 compared to 14/16 among single participants).

The narrative below draws out recurring themes and sub-themes from the 22 participants with experience of online partner-seeking, structured around the three elements that comprise a social practice (meanings, materials and skills) with a summary of all themes given in Table 2. We describe how meanings were understood in terms of online partner-seeking being a normalized practice, with a multiplicity of goals, balancing possibility and risks, with withdrawal symbolizing commitment. We describe materials in terms of the affordances of the technologies used for online partner-seeking. We consider competencies in terms of technical competencies, and social and emotional competencies.

**Table 2.** Themes and sub-themes.

Theme	Sub-themes		
Meanings	Normalized practice Enmeshment within broader online cultures of image presentation		
	Multiplicity of goals Balancing possibility and risk	Meeting partners for enduring romantic relationships Meeting people for one-off sexual encounters Dating Boosting self-esteem and empowerment Entirely online sexual actions and interactions Sharing online experiences with friends	
		Possibilities Risks	Enabling sexual adventure Instrumental efficiency Boredom Presenting oneself as “sleazy” Self-loathing Rejection Misaligned expectations Unsolicited explicit images or premature or otherwise inappropriate explicit proposition False representations Meeting “creeps” or the risk of abuse or violence Onward sharing of explicit images
	Withdrawal from online dating symbolizing commitment		
Materials Competencies	Affordances of the technologies Technical competencies	Efficient searching and screening and blocking of potential contacts Range of different apps and websites General social media technologies Blurring of online and face-to-face networks and interactions Searching for and assessing potential contacts Creating and sharing intimate images to minimize risks that these would be shared	
	Social and emotional competencies	Managing one’s interactions with contacts Self-care	Being clear about whether their goals aligned or not Being clear with oneself about one’s goals Setting limits to one’s engagement

## Meanings

Online partner seeking was imbued with various meanings which linked to participants’ actions and attitudes.

### Normalized Practice

Participants commonly regarded seeking partners for sexual encounters as a normalized part of broader online and sexual practices in contrast to its previously marginalized and stigmatized status. However, experiences of and attitudes to the practice varied. The practice was much more normalized among younger people and was particularly central to the life of many sexual minority participants. One participant described how the practice quickly became normalized in her own life:

So, yeah, obviously five years ago, I don’t even know if Tinder was around. So I came out of my relationship in January and I got Tinder and I’m on Hinge now and it’s a whole new world. It is, it’s mind-boggling because I’d never thought I would go on a dating app. Never ever thought it, I thought it’s for saddo people. And it’s not, everyone seems to be on it.

(female, 20s, white British, single, bisexual)

Despite this normalization, participant accounts suggested that there often remained a sense of discomfort with, or ambivalence about, some aspects of the practice within people’s intrapsychic sexual scripts. In particular, some participants suggested that the practice could feel too “clinical” or cold. This varied and was less apparent among men and sexual minority participants. For example, one straight woman who used dating apps described her discomfort with some aspects of online dating.

I just couldn’t imagine like sitting there arranging to meet someone for sex and then like … organising a meeting at work, it’s just too like clinical.

(female, 30s, white British, single, heterosexual)

### Enmeshment within Broader Online Cultures of Image Presentation

Several participants viewed online partner seeking as being one element within broader online cultures of self-presentation. Participants described how this could enable self-expression, self-experimentation and self-validation. But some also described how this could lay one open to critical judgment, and could contribute to a culture of superficiality and unrealistic expectations about bodies and lifestyles. One woman commented:

You’re seeing more people, for Instagram, for example, people in bikinis, you know, seeing people who are physically fit. And people more want that … Like you want to try and make yourself look better … I think it’s a bit sad really, that people are doing that, and people don’t feel like they’re good enough because of the photos, and it’s not even real … I think there’s a lot of expectations that aren’t real and I guess it’s a bit like porn as well, for guys. They expect that’s what it’s going to be like but it’s not.

(female, 20s, white British, single, bisexual)

### Multiplicity of Goals

Central to the meaning of online partner seeking for participants was the multiplicity of goals involved, which was not limited merely to meeting partners for face-to-face sex and/or relationships. The goal of *meeting partners for enduring romantic relationships* was an uncommon theme in the accounts of many participants regardless of gender or sexual orientation. This was almost never mentioned by participants, which may have reflected their goals, their focus on the most immediate short-term goal or their desire within the interview to present themselves as focused on fun rather than a committed relationship. Participants more often identified the goal of *meeting people for one-off sexual encounters* (often termed “hook-ups”). One man described the immediacy of this goal:

I don’t really do the dating stuff because historically when I use these apps I’m already in a certain of frame of mind where I’m wanting - usually - sex … and I’m not usually, you know after a few drinks I’m horny and not really wanting, I’m not really satisfied then with making a meet for next Tuesday … for a beer because it doesn’t … it doesn’t tick it for me

(male, 30s, white British, single, gay)

Participants sometimes mentioned *dating* as a pleasurable goal in itself, regardless of whether this led to sex or a relationship. One man emphasized the social aspects of dating:

But I like the, I like, dating I think is good fun, it’s really good, and I kind of got, you know, maybe into the habit of going for the sake of dating …

(male, 30s, white British, heterosexual, co-habiting)

Participants also identified *boosting self-esteem* and *empowerment* as important goals, again regardless of gender or sexual orientation. They linked this to receiving affirmation either during online or subsequent face-to-face interactions, exemplified by this young woman’s account:

And they would always call me beautiful and things like that and I enjoyed that … I enjoyed … I enjoyed being called nice things because obviously … my partner before wasn’t very nice towards me … I, I think I enjoyed people saying they would want to sleep with me … but I never did anything with them

(female, 20s, white British, cohabiting, heterosexual)

Some participants discussed how they, or others they knew, sometimes engaged in entirely online sexual interactions with no interest in seeking partners for face-to-face sexual encounters or intimate relationships. Such online activities could include scrolling and browsing to check out who was online, exchanging flirtatious or suggestive messages and exchanging images or videos. This was apparent as a theme in the accounts of participants across gender and sexual orientation. One man’s account resonates with the finding of previous research that online searches can highlight the presence of potential contacts in one’s local area (Albury & Byron, 2016).

Yeah I’ve used [Tinder] but just for like, just for fun like … Just like, just message girls, just like chat to them and I don’t know, see like if I could see people I know … Like honestly just for fun, like I’m bored.

(male, <20, white British, single, heterosexual)

Misaligned goals led to frustration, such as when one partner sought a physical meeting and the other online interaction only, or when one sought immediate contact and the other delayed. A final goal expressed by participants was *sharing online experiences with friends* for fun or to give an insight into each other’s lives:

Yeah, my best friend, she’s been single for a bit, so sometimes she will, I’ve got Plenty of Fish app, I’ll be like, “come on, let’s have a look, let’s see who you’re talking to”, just as a bit of a joke and stuff, so it’s a bit of fun between us.

(female, 20s, white British, cohabiting, heterosexual)

### Balancing Possibility and Risk

A key meaning underlying the practice was the balance between increased possibilities and managing risk. Participants accepted this balance as integral to the engagement, regarded it as a source of ambivalence, or found it motivated their disengagement from the practice. Participants raised a number of *possibilities*. A few participants, in particular straight or gay men, presented the practice as *enabling sexual adventure* and new experiences:

I guess digital media gives you the options to have experiences that you might not, potentially, get to experience.

(male, 30s, white British, heterosexual, co-habiting)

However, a much more common theme among men and women of different sexual identities was the *instrumental efficiency* of the practice in enabling easier contact with potential sexual or romantic partners. As earlier research has suggested, an economic logic appears fundamental to intrapsychic scripts relating to online partner seeking. For sexual and religious minorities in particular, use of specific apps was regarded as critical to maximize the ease and efficiency of meeting appropriate partners, as this quote from a women with previous experience of same-sex relationships reported:

I think for a gay woman it’s just easy, because you know that, they’re specifically saying they’re gay, there’s no sort of hidden, you know, I’ve had that before, in a club when I was younger, talking to a girl and then … find out she’s straight, or she’s, you know, not into women or whatever, and it’s a bit like, oh God.

(female, 20s, white British, cohabiting, heterosexual)

Heterosexual people also saw dating apps in economic terms. They could increase the effectiveness and efficiency of meeting partners, partly through being able to view so many potential contacts and partly, when using certain apps, being able to identify partners with particular characteristics:

It’s one of those ones the way kind of technology has advanced that’s allowed that, erm, the kind of days where you needed to go to a pub, a club, randomly kind of bump into someone there, you’re meeting someone for the first time drunk usually.

(male, 30s, white British, co-habiting, heterosexual)

As well as possibilities, participants identified a number of *risks* that were important to how the practice was understood. In contrast to previous research (Couch et al., 2012), these risks often related to one’s own actions or feelings rather than to the actions of others. *Boredom* was a very commonly reported central feature of the practice. This could arise as a result of investing time in boring interactions or as a result of not limiting one’s engagement in online scrolling of potential contacts. One man described the pointlessness of extended online interactions:

I just found it quite like, I don’t know, I found it quite boring actually, just, like, some, I would try conversations and I thought to myself this is a bit rubbish like you just, I think I would, if I was to use it again I would actually strike up a conversation and very quickly ask them would you like to meet up …

(male, 20s, white British, single, heterosexual)

Boredom also included complaints about the mundanity of the practice from a few gay as well as straight participants, whereby online dating was viewed as less, not more, exciting than face-to-face contact.

It can be a bit of a, bit of a soulless experience

(male, 30s, white British, single, gay)

It’s a strange thing, when you sit down and think about what Tinder is, I mean, it takes away some of the nervousness, but also takes away some of the excitement. I mean, it’s not so much fun saying … “I’m just chatting to this person online”.

(male, 30s, white British, heterosexual, co-habiting)

Another recurring theme was that one’s engagement in the practice risked inadvertently *presenting oneself as “sleazy”* particularly when using apps associated with hookups. One gay man described how this might hamper the ability of those searching for an enduring relationship to enact the appropriate sexual script for an encounter:

I suppose the sleaziness of it … I think it’s, it’s known for that as well. So I think sort of in terms of image then, if you’re on there and sort of looking for a boyfriend, which I actually wasn’t but say I was, you were on there looking for a partner, I mean you’ve got a bit of an image problem

(male, 20s, white British, non-cohabiting relationship, gay)

Another risk raised across participants’ accounts was a sense of *self-loathing* that could arise from the practice. This was associated with negative feelings about the superficial attitude to people that one could develop, or with regret about one’s interactions with other people, often under the influence of alcohol or recreational drugs. One gay man described the former and how this linked to the rapid screening of potential contacts:

There’s Tinder, Grindr and Plenty of Fish, they’re the three apps that I have had, deleted, had, deleted, had, deleted, had, deleted, had, deleted, had. And you go through it all the time … I was never really keen on it because it sort of makes you, it sort of makes you feel quite bad about yourself at times when you’re just swiping and you’re like “Oh my God, how many people am I going to go through?” And then you think what am I actually looking for because I’m just looking at this man’s face and that’s it … It doesn’t tell me anything.

(male, 30s, white British, single, gay)

Relatedly, a few participants identified *rejection* as a risk, for whom a sudden and unexplained ceasing of communication could cause emotional upset, as illustrated by this woman’s account:

Sometimes conversations go on for weeks and there will be endless texting, then you never end up meeting up with them or they’ll just disappear and like never message you again and you don’t know why.

(female, 30s, white British, single, heterosexual)

Participants identified *misaligned expectations* as risks that could derail interactions by confusing what sexual scripts were being enacted. This was clear from the accounts of some participants across gender and sexual orientation. This could involve, for example, one person wanting a sexual encounter while another sought dating or merely online flirting:

A lot of people … seem to be content that that’s their meet and it’s like you’re chatting and you think it’s going to lead to a meet … and it’s like “Should we Skype?” or “Send me a picture” or “Should we chat … a video chat?” And I’m like “No, we don’t, that totally does not do it for me.”

(male, 30s, white British, single, gay)

A very common risk reported by many participants was *receiving unsolicited explicit images* or *premature or otherwise inappropriate explicit propositions*. As the quotes below illustrate, this was more often reported by women and gay men, and presented as a very common annoyance or a turn-off rather than as a source of more serious upset.

On Grindr a lot of, a lot of unwanted attention definitely … it would be like … I’d get messages like … “I would pay you to poo on me” … And people would send pictures of poo. People would send like, you know, money signs, like oh, you know “I’ll give you £200 if you … ” I distinctly remember somebody wanted me to come to like a sex party and, you know, essentially acting like a, a piece of meat for lots of different men.

(male, 20s, white British, non-cohabiting relationship, gay)

Like you’ll start a conversation, be like “Hi, how are you?” … And the next time you’ll get “I’m horny”. What do you want me to do with that bit of information? … As soon as like someone mentions sex, like and I’ve not even spoken to on the phone or met you, it puts me off.

(female, 30s, white British, single, heterosexual)

Female participants also commonly reported encountering or being aware of contacts who provided *false representations* of themselves, which was presented as a more serious risk because of its potential association with personal danger.

Like you can get catfished, you think you’re talking to this person who’s put photos up of somebody else. You don’t even know if that is them until you meet up with them.

(female, 30s, white British, non-cohabiting female partner, bisexual)

Several women reported *meeting “creeps” or the risk of abuse or violence* as significant sources of risk. For one woman this was a factor behind her disinvestment from the practice:

It’s pure creeps, absolute creeps. Basically, like, I think social media’s just a platform for idiots and deviants. And there’s people out there, you’ll get messages off lads and you think, “Oh God, have you actually sent that message to me?” But then you take a step back and think, “Oh, my God, there’s actually lasses out there that reply to these type of messages, which are making these imbeciles think that it’s OK to speak to women like that.

(female, 30s, Asian British, single, heterosexual)

Another significant risk reported by participants, regardless of their gender or sexual orientation, was *onward sharing of explicit images* of themselves without their consent. How participants managed this risk is discussed later under “skills.”

Um, they could then sort of be available for wider sharing, um, which I think was quite common … Um, people I didn’t want to see it … you never know who’s going to see.

(male, 20s, white British, non-cohabiting relationship, gay)

### Withdrawal from Online Dating Symbolizing Commitment

A final meaning, resonating with earlier research (Albury & Byron, 2016) on online partner seeking, was withdrawal from the practice symbolizing commitment to a monogamous romantic relationship. Regardless of gender or sexual orientation, participants reported removing apps or sharing social media accounts as signifiers of new commitments:

I think I deleted the app off my phone because we had exchanged numbers on the dates we were on …

Interviewer: So by that time you were just texting?

We were texting, yeah.

(male, 30s, white British, co-habiting, heterosexual)

## Materials

Participants described how their experiences and attitudes about online partner seeking were influenced by technologies. In particular, participants described how the *affordances of the technologies* employed partly shaped their online partner seeking practices. A key aspect of the technologies used for online partner seeking was how they facilitated *efficient searching and screening and blocking of potential contacts*. Some individuals emphasized keeping one’s options broad and using rapid screening to view all local potential contacts. One gay man described how the lack of filters used in his searches related to his goal of seeking a hookup rather than a more enduring relationship:

I just tend to keep it based on the GPS which is your 100 nearest profiles to wherever the application is.

(male, 30s, white British, single, gay)

Other participants, such as the participant below, emphasized use of filters to try to ensure the most potentially compatible contacts would be presented:

I mean you can slide your filters if you want Mr Right or Mr Right Now.

Erm, it was on I think that was on Tinder.

(male, 20s, white British, single, gay)

A straight man described how he used a more specific search strategy to better define his interests:

Plenty of Fish is kind of more, was one of the first ones, so it’s kind of similar to Match but free … You basically you can put filters on to say “I’m looking for someone within this kind of radius, this age range.” I think you can probably do hair choices and stuff, things that they like. And then it would give you like kind of a list and you could look through profiles and see what people wrote about themselves

(male, 30s, white British, co-habiting, heterosexual)

Several women and gay men emphasized the importance of blocking functions which afforded the termination of abusive or other unwanted interactions. As the quote below illustrates, different apps offered different blocking affordances:

On Grindr if there was someone you didn’t like, you’d have to block them … And there would also be restrictions on the number of people you could block … per day. Um, whereas on Tinder, you know, it’s just a quick left and then they’re gone

(male, 20s, white British, non-cohabiting relationship, gay)

Building on this point, a recurring theme across all demographic groups was that there was a *range of different apps and websites* which were available to enable partner seeking. These afforded access to different social networks/audiences, as well as different modes of communicating (for example, moving from text to voice to video). Participants of varying age, gender and sexual orientation described certain apps such as Tinder and Grindr as primarily facilitating hookups linked to their focus on screening contacts primarily via visual cues.

One apps for one thing, one’s for another, one’s for another. And I doubt anybody that pulls on Grindr in their lunch break’s looking to find a boyfriend or a husband.

(male, 30s, white British, single, gay)

There’s no beating about the bush: Tinder is out for what I think is, it’s just a hook-up app, and I used it exactly for that.

(male, 30s, white British, heterosexual, co-habiting)

Other apps were described as more commonly used to seek partners for more enduring relationships, linked to their greater filtering of contacts based on characteristics such as personality, interests etc.

[With] Plenty of Fish, there’s a mix of … wanting to look for somebody because there’s pictures up and you’re giving them all the details about you.

(male, 30s, white British, single, gay)

A novel finding was that a large number of participants reported using *general social media technologies*, such as Facebook, Instagram and online gaming rather than dating-specific technologies, to meet partners. Younger and heterosexual participants most commonly reported this. It was not reported in the accounts of those seeking same-sex contacts, for whom dating-specific apps’ abilities to target contacts based on sexual preference were a key affordance. As well as using generic rather than dating-specific technologies, a recurring theme in accounts from younger heterosexual participants was meeting contacts via a *blurring of online and face-to-face networks and interactions*.

She was friends with, like, loads of my friends and then … I think … I followed her on Instagram. Like, my friend must have posted a picture of her, then I followed her and then like tried to like meet, like flirt with her …, move with, get with her.

(male, <20, white British, single, heterosexual)

## Competencies

Participants referred to various skills which, alongside technological affordances, influenced their online partner seeking.

### Technical Competencies

Some of these skills were technical, concerning how participants were adept in using the technology to efficiently achieve their goals while minimizing the risks of unwanted consequences. Participants very commonly described how they developed and deployed a range of technical skills in *searching for and assessing potential contacts*, which are described above.

Earlier, we reported participants’ concerns about unconsented onward sharing of explicit images of them by contacts. Participants described technical skills they employed in *creating and sharing intimate images to minimize risks that these would be shared* or, if shared, that would identify them. One gay man described preventing sharing via use of appropriate technologies:

if you’re sending a picture on Grindr then it’s a picture they could keep and potentially share, whereas if you’re sending it on Snapchat it’s … Yeah. There was, yeah, there was an element of concern, um, more with like face pictures as well, you know, full, fuller pictures, I’d prefer that they were on Snapchat because they can’t sort of re-see them or share them.

(male, 20s, white British, non-cohabiting relationship, gay)

One straight woman described not including her face in such images so that even if shared these would not identify her:

Yeah. I have … I did send a few images but it was still just my breasts.

(female, 20s, white British, cohabiting, heterosexual)

### Social and Emotional Competencies

As well as technical skills, participants described several social and emotional skills they used to facilitate their satisfactory practice of online partner seeking and minimize some of the risks described above. A core set of skills were social skills in *managing one’s interactions with contacts*. Key among these was clarifying goals so that both parties to an interaction were *clear about whether their goals aligned or not*, so as not to waste each other’s time as described earlier. These were most salient in men’s accounts. One man described how he quickly and assertively defined his intentions so that the other person could determine if their goals aligned:

It’s something you can establish quite early on Tinder, where you can literally just say, “Are you looking for a relationship or are you looking for just hook-ups? Because more often than not, if you say hook-ups and they don’t like it, they just delete the chat, you delete the chat …

(male, 30s, white British, heterosexual, co-habiting)

Another set of key skills, relating to intrapsychic rather than interpersonal sexual scripts, was in *self-care* to minimize emotional harm arising from one’s engagement in the practice. One such self-care skill was *being clear with oneself about one’s goals*:

I’m fairly, eh, wise, as most men are, to what these sorts of apps can bring, so as long as you’re well informed and you’re not too easily led then … it does, it does what it … says on the tin so to speak, yeah.

(male, 30s, white British, single, gay)

Another such skill was in *setting limits to one’s engagement* in online partner seeking to avoid committing too much of one’s time and emotional energy. One man described how he regulated the time he spent and did not use it as a means of killing time:

I tend to only use them if I’m looking to meet someone … I wouldn’t, I don’t browse. I’m looking for something specific i.e. a meet so then that would be an informed decision, I don’t just browse through it …

(male, 30s, white British, single, gay)

## Discussion

### Summary of Key Findings

Our study is the first qualitative study to examine different aspects of online partner seeking among a population diverse in terms of gender, age, sexual orientation and technologies used. Reading online partner seeking through the lenses of sexual scripts and social practices enabled us to view this as an individually enacted but socially defined practice imbued with shared meanings. It is a practice facilitated by the material affordances of the technology as well as the technical and social and emotional skills employed by its users. These skills enable users to achieve their diverse goals while protecting themselves from various potential harms. Like previous research on this phenomenon, our findings emphasize the affordances of the technology and how these enable or constrain the experiences and actions of users (Christensen, 2020; Degen & Kleeberg-Niepage, 2020) but we note the importance of user strategies and skills in creatively choosing between and shaping use of technology toward diverse goals.

Within participants’ intrapsychic sexual scripts, online partner seeking was viewed as a normalized social practice (particularly among younger and sexual minority individuals) yet one widely regarded with ambivalence. Users identified a range of meanings, central to which was balancing different goals and risks. In line with previous studies, these goals included seeking purely online sexual interactions, noting local users (Albury & Byron, 2016), bolstering self-esteem (Ward, 2017) and not merely meeting partners for face-to-face sexual encounters or relationships. In fact, seeking relationships was rarely mentioned as a goal by any participants. It may be that this reflects a residual stigma associated with seeking partners online so that while users will admit to seeking fun and casual sex they are less willing to admit to seeking more committed relationships online. Or it may simply be that participants viewed the immediate goals as casual encounters or relationships, and viewed more enduring relationships as distal possibilities that are not foregrounded in their accounts. Rather than providing excitement and adventure, meeting partners online was more often viewed as instrumentally efficient, in line with some previous literature emphasizing the economic logic of meeting partners online (Degen & Kleeberg-Niepage, 2020). As reported previously (Albury & Byron, 2016), for those who developed enduring relationships through online dating, deleting apps and disengagement from the practice could symbolize commitment.

Our study provides some novel contributions to the existing literature. In contrast to previous research (Couch et al., 2012), we found that many risks were defined not merely in relation to “risky others” but actually in terms of one’s own actions or attitudes, such as: boredom; “soulless” experiences; self-loathing associated with the superficiality of online interactions; regrets; and self-presentation as “sleazy.” Again, this resonates with the idea that participants apply an economic logic to the practice (Degen & Kleeberg-Niepage, 2020), so that one’s own conduct is assessed in terms of whether it represents a wise investment.

We also identified a complex range of intersecting technical and social/emotional competencies. A novel finding was that the accounts of young heterosexual people commonly cited general social media apps rather than dating apps, and meeting partners often involved a complex interplay between online and offline networks and encounters, reflecting the pervasive role of social media in young people’s lives. Sexual minority participants were much more likely to choose dating-specific apps, particularly those designed for their communities. They favored such apps because these facilitated efficient screening of a large number of potentially appropriate contacts. Technical competencies included skills in searching and screening but also skills in blocking unwanted contacts and in minimizing the risk of consequent harms of non-consented onward sharing of intimate images, which has been raised in previous literature on sexting but not online dating. Previous research has suggested that gay men commonly apply skills in brief communications to rapidly move toward hookups, whereas heterosexuals are more likely to use more involved communications to establish compatibility (Licoppe, 2019). However, we found that accounts of communication styles and goals did not map neatly with sexual orientation.

A further novel finding was the range of social/emotional skills featuring in participant accounts, including those relating to self-care, setting limits to engagement in online partner seeking and self-awareness of one’s goals in engaging with the practice. Whereas previous research has emphasized users’ reference to rules in terms of ensuring physical safety (Albury & Byron, 2016), we found evidence for rules largely focused on self-discipline and clarity of goals.

### Strengths and Limitations

This study drew on a broad sample diverse in terms of geography, gender and sexual orientation to explore online partner-seeking as a broad set of social practices. A limitation of our study is that participants were not purposively selected on whether they had sought partners online so only 22 of the 40 people we interviewed could discuss the subject (with two engaging in the practice once, eight occasionally and 12 more often). This was particularly so among older participants and those of ethnicity other than white British, despite the sampling encompassing age and ethnic diversity. We cannot ascertain from our qualitative data whether this under-representation in our analytic sample reflects a lower rate of online partner seeking among these groups or merely the chance composition of our sample. Furthermore, reflecting the sample, our results refer only to certain apps and hence our findings may not be generalizable to other technologies. Nonetheless, more than half of the people we interviewed did have direct experience of seeking or interacting with potential partners online, and participants were from across Britain, were diverse in age and sexual orientation, and had used a variety of digital platforms. This heterogeneity contrasts with much of the previous research that has tended to focus on specific sub-groups or users of particular apps.

### Implications for Policy and Research

The public health literature suggests that those seeking partners online may be more likely to engage in sexual risk behavior (Cabecinha et al., 2017) and considers dating apps as potential sites or tools for promoting sexual and mental health among groups such as young people and men who have sex with men (Ems & Gonzales, 2015; Holloway et al., 2014; Rendina et al., 2013). Our participants did perceive themselves to encounter various risks through seeking partners online, often centered on threats to their emotional health, but these did not include involvement in sexual risk behavior. Our participants used a number of technical, communication and self-care strategies to manage risks. This suggests that some who seek partners online will welcome health promotion interventions which address the multiple risks that users can experience. A focus on social practices encourages interventions to engage with what practices mean, and how meanings or competencies might be influenced in order to reduce risks (Blue et al., 2016). Such interventions need to recognize the meanings and motivations underlying online partner seeking, and build on users’ existing competencies. Informed by our findings, e-health interventions or school relationships and sex education might, for example, support people in developing and implementing clear plans about: their intentions and how these align with their broader goals; what behaviors they will and won’t engage in; what technologies they will use and how they will use these to meet partners; how they would respond to abusive or unwanted communications; and how to maintain self-esteem. Our research suggests the importance of interventions recognizing that the social practice of online partnering is not confined to dating apps.

Regarding further research, while our findings have emphasized the commonalities of experiences among our participants, it has also suggested some distinctions by age, gender and sexual orientation, for example, in the goals of seeking partners online, the technologies used and experience of unsolicited images or propositions. Further research is needed to explore these distinctions using purposive samples, as is more research on seeking partners online among gender diverse and ethnic minority populations.

## Supplementary Material

Supplemental Material

## Data Availability

We are archiving anonymised transcripts with the UK data service to be available to researchers after completion of the Natsal-4 study.
