# Supplementary material for: Online Sexual Partner Seeking as a Social Practice: Qualitative Evidence from the 4th British National Survey of Sexual Attitudes and Lifestyles (Natsal-4)
Source: J Sex Res. 2021 Nov 18;59(8):1034–44. doi: 10.1080/00224499.2021.1994516 (PMC9519119; doi:10.1080/00224499.2021.1994516)
Supplement: Supplemental Material [file HJSR_A_1994516_SM7045.docx]

**Appendix: Interview Topic Guide**

**Background**

In your own words, can you tell me a little bit about yourself? *[Probes: where grew up, where live, family life, school/work, friends?]*

Please tell me how you identify in terms of your gender and sexual identity.

Please tell me about your current or most recent sexual or intimate relationship. Who is/was it with? How did you meet them? How would you describe this relationship?

Please tell me about your previous sexual or intimate relationships. Were these with same- or other sex partners? About how many partners?

| **Opening questions**  What part do digital media play in your life?  What role do digital media play in your current and previous relationships?  Other than meeting partners, do you use digital media to get to know people who share the same gender or sexual identity as yourself?  Do you look at sexual images, videos or other sexual content?  Have you ever experienced abuse linked to the use of digital media in sex or a relationship? | **Follow-on probing questions**  How do you access the internet and what do you use it for?  What apps do you use?  How do you use text messaging?  Have you used digital media to meet partners?  Have you used digital media  in a sexual way with partners, such as sending and/or receiving sexual text messages, pictures or videos, or having sexual encounters using phone or via video link?  If so, in each case what was exchanged in terms of contents?  In each case, did you send and/or receive?  How do you do this?  Do you use specific websites or apps etc.?  What experiences have you had doing this?  How have these made you feel?  What benefits or problems have you experienced doing this?  How do you access these?  What do you look at or read? And why?  How often do you access these?  How does looking at this material this make you feel?  Is this something you do on your own or with others?  Have you ever been abused by a partner using digital media, such as verbal insults or threats, or a  partner using digital media to pry on or try to control your movements?  Have you ever abused a partner using digital media, e.g., verbal insults or threats or using digital media to pry on or try to control a partner’s movements?  Has anyone ever shared a sexual message, image or video of/from you with other people? Without your permission?  Have you ever shared a sexual message or image of/from someone you know? Without their permission? | **Further probes**  How do you use these media for interaction with others?  How much time per day do you spend using these media?  When in the day and in the week do you use them most?  If so what type of media, sites or apps did you use and how did you use them?  If so please describe this.What were your motivations for this?  Describe what this involves.  What type of technology, websites or apps were used?  What experiences have you had doing this?  How have these made you feel?  What benefits or problems have you experienced doing this?  Phone? Computer? Television? Books? Magazines?  Arousal? Masturbation? Curiosity? To learn about sex?  Are there particular times in the day or the week?  What are the positives for you?  What are the negatives for you?  Partners? Friends? Colleagues? Anyone else?  If so please describe this and how it made you feel.  If so please describe this.  If so please describe this and how it made you feel.  If so please describe this. What was your motivation? |
| --- | --- | --- |

**CONCLUSION**

1. Is there anything else you’d like to tell us about that you think we should be thinking about in relation to this?
2. Do you have any questions for me at this time?
